# Supplementary figures and images for: BCC0 collaborates with IMC32 and IMC43 to form the Toxoplasma gondii essential daughter bud assembly complex
Source: PLoS Pathog. 2024 Jul 18;20(7):e1012411. doi: 10.1371/journal.ppat.1012411 (PMC11288415; doi:10.1371/journal.ppat.1012411)

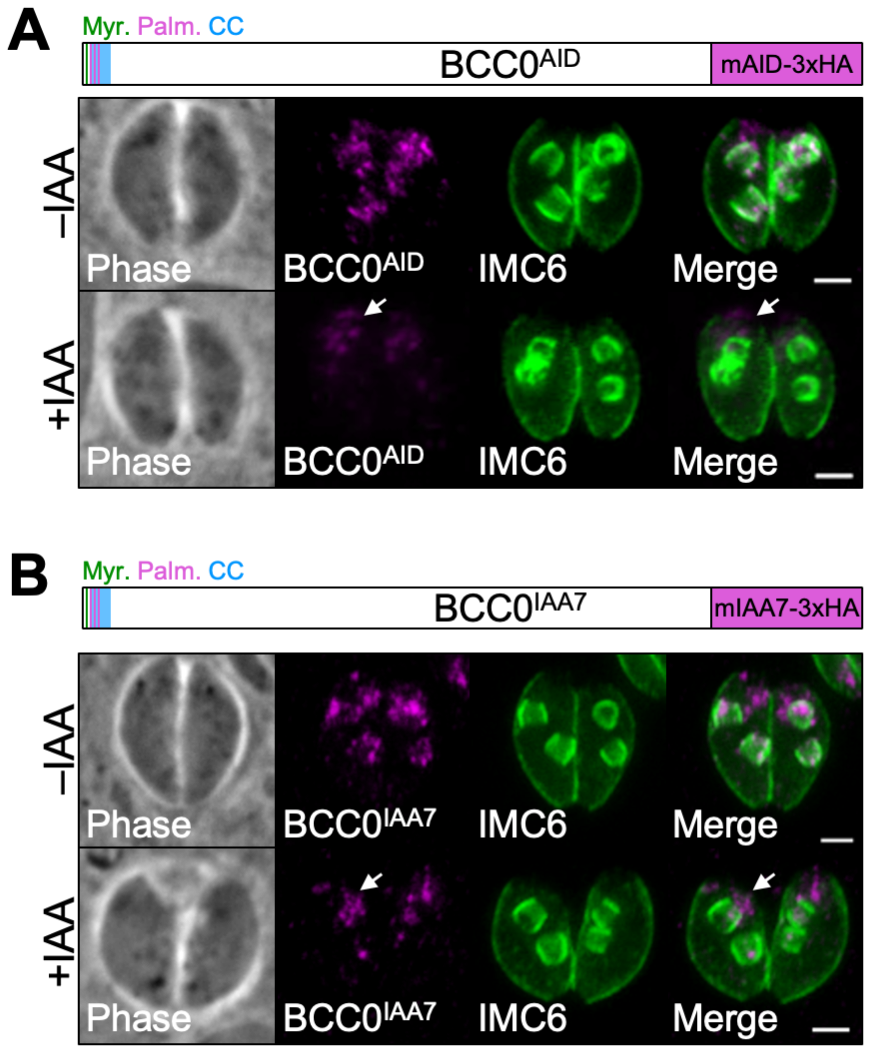

Supplement: S1 Fig — A) Diagram and IFA of BCC0AID parasites grown for 24 hours -/+ IAA showing that BCC0AID does not provide sufficient protein knockdown (arrow). Magenta = anti-HA detecting BCC0AID, Green = anti-IMC6. B) Diagram and IFA of BCC0IAA7 parasites grown for 24 hours -/+ IAA showing that BCC0IAA7 does not provide sufficient protein knockdown (arrow). Magenta = anti-HA detecting BCC0IAA7, Green = anti-IMC6. Scale bars = 2 μm. (TIF) [file ppat.1012411.s001.tif]

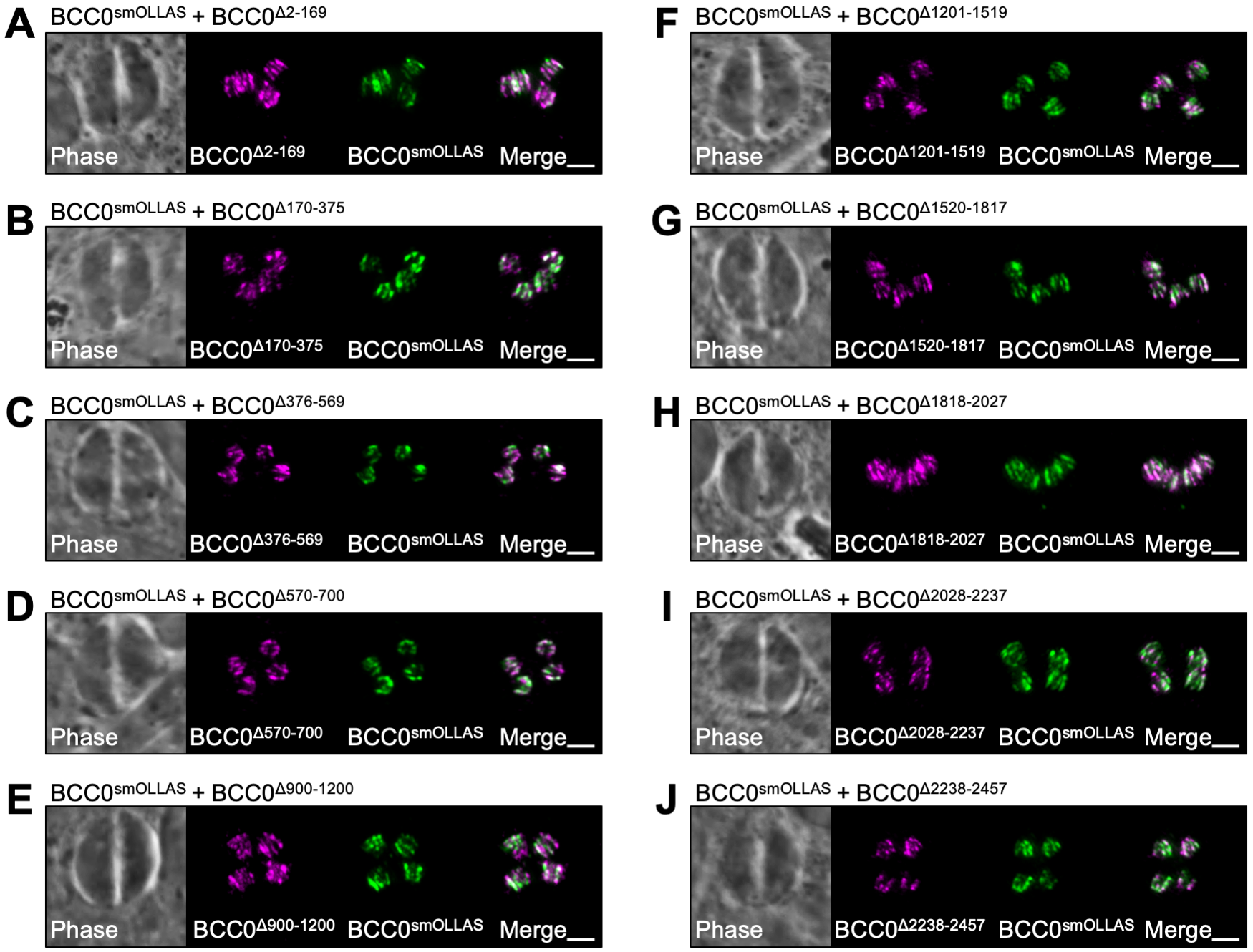

Supplement: S3 Fig — IFAs showing that all BCC0 deletions except for BCC0Δ701–877 (shown in Fig 4D) colocalize with endogenous BCC0smOLLAS. Magenta = anti-HA detecting smHA-tagged BCC0 deletion constructs, Green = anti-OLLAS detecting endogenous BCC0smOLLAS. Scale bars = 2 μm. (TIF) [file ppat.1012411.s003.tif]

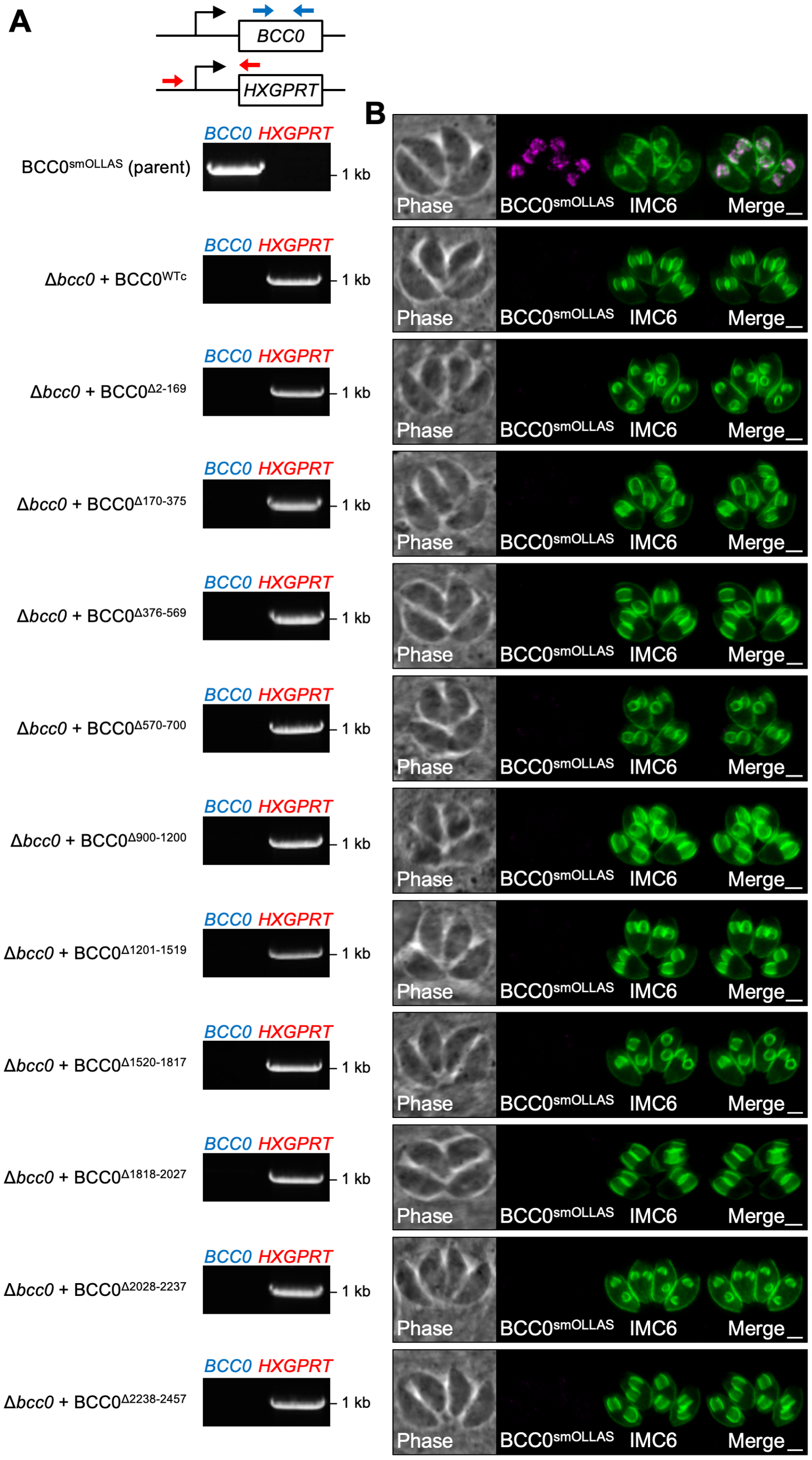

Supplement: S4 Fig — A) PCR verification for genomic DNA of BCC0smOLLAS (wild-type parent strain) and complemented Δbcc0 parasites. Diagram shows the binding location of primers used to amplify the BCC0 coding sequencing (blue arrows) and the site of recombination for the knockout (red arrows). The strain used in each PCR verification is indicated on the left of each image. B) IFA of complemented Δbcc0 parasites confirms loss of BCC0smOLLAS signal. Each IFA in panel B corresponds with the PCR verification to the left of it in panel A. Magenta = anti-OLLAS, Green = anti-IMC6. Scale bars = 2 μm. (TIF) [file ppat.1012411.s004.tif]

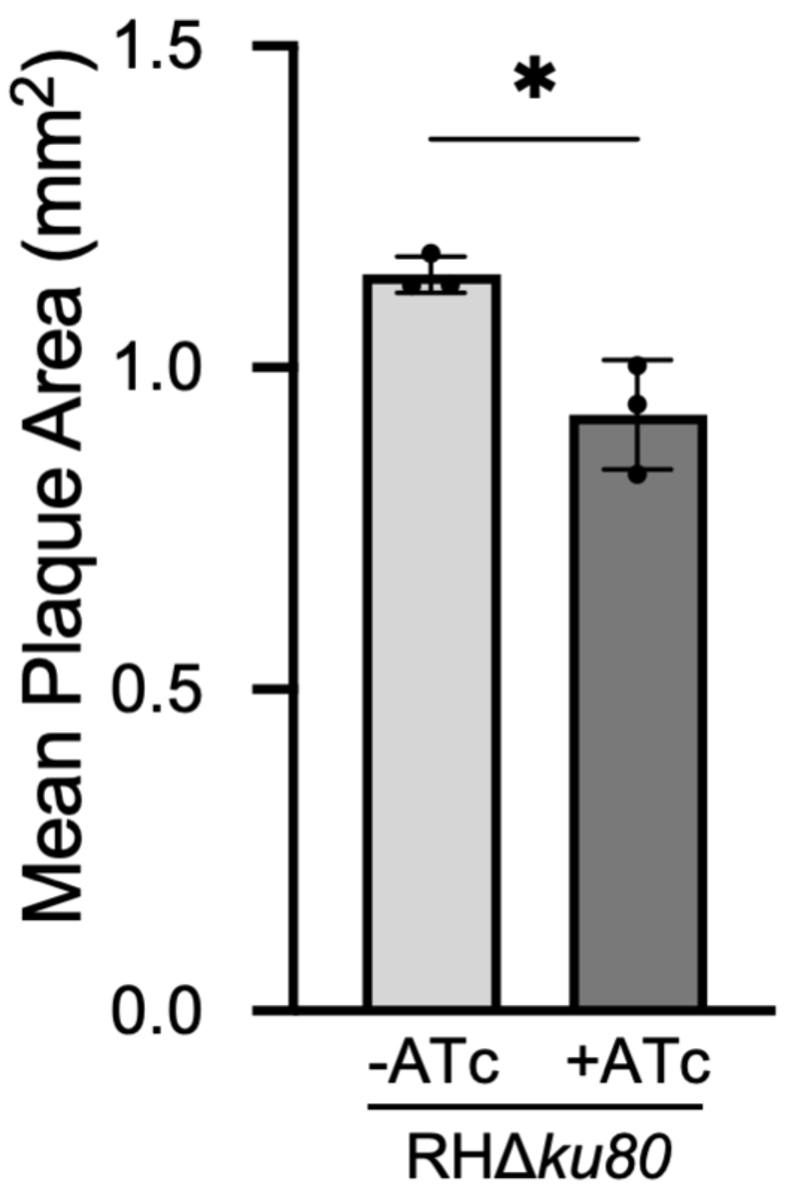

Supplement: S5 Fig — Quantification of plaque size for RHΔku80 parasites grown for seven days -/+ ATc. Statistical significance was determined using a two-tailed t-test (*, P < 0.05). (TIF) [file ppat.1012411.s005.tif]

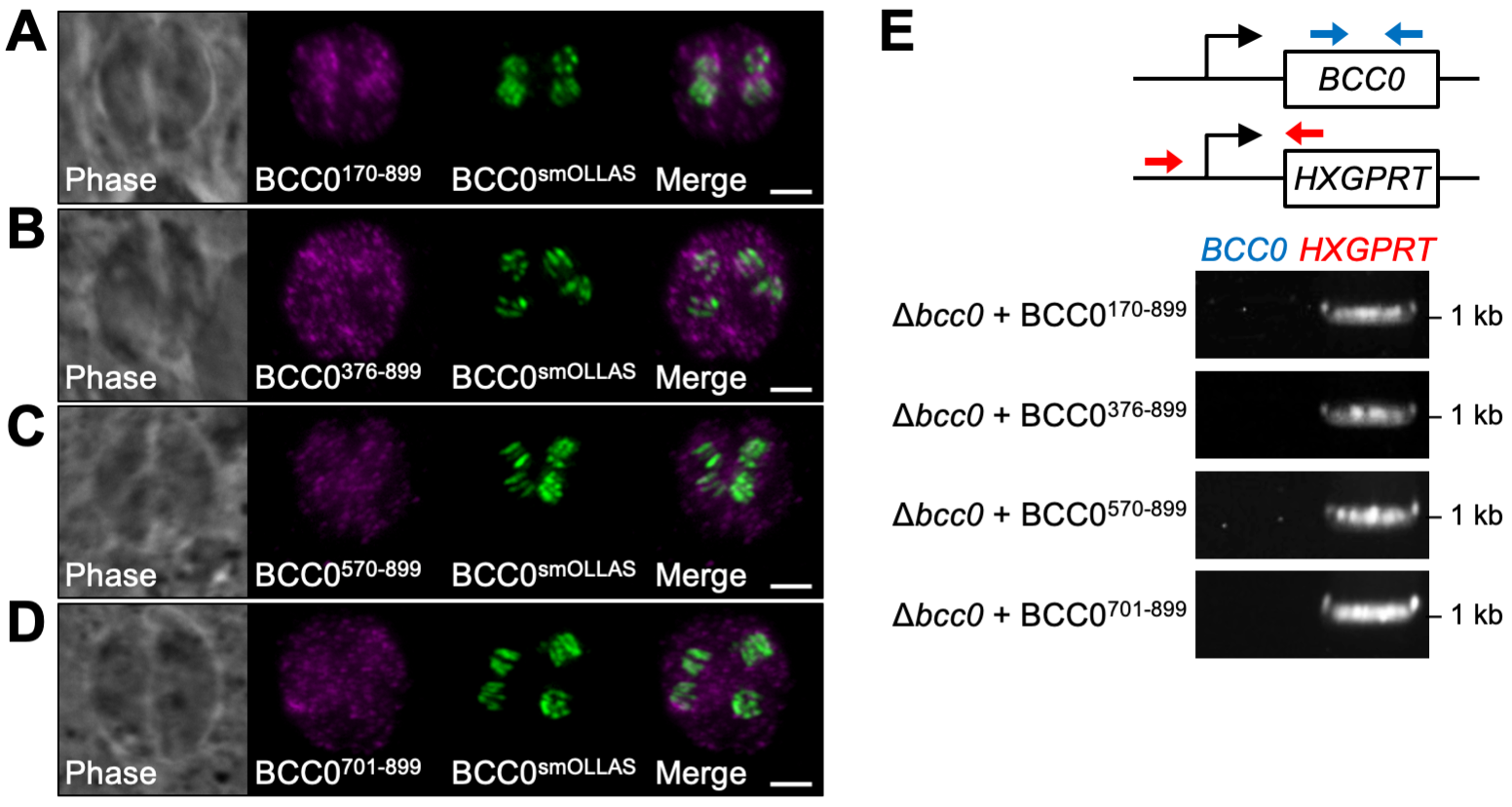

Supplement: S6 Fig — A) IFAs showing that BCC0170-899, BCC0376-899, BCC0570-899, and BCC0701-899 all mislocalize. Magenta = anti-HA detecting smHA-tagged BCC0 deletion constructs, Green = anti-OLLAS detecting endogenous BCC0smOLLAS. Scale bars = 2 μm. E) PCR verification for genomic DNA of complemented Δbcc0 parasites. Diagram indicates the binding location of primers used to amplify the BCC0 coding sequencing (blue arrows) and the site of recombination for the knockout (red arrows). A control PCR verification performed on BCC0smOLLAS (wild-type) parasites can be seen in S4A Fig. (TIF) [file ppat.1012411.s006.tif]
