# Supplementary material for: BCC0 collaborates with IMC32 and IMC43 to form the Toxoplasma gondii essential daughter bud assembly complex
Source: PLoS Pathog. 2024 Jul 18;20(7):e1012411. doi: 10.1371/journal.ppat.1012411 (PMC11288415; doi:10.1371/journal.ppat.1012411)
Supplement: S2 Fig — The amino acid sequence of BCC0 (TGGT1_294860) was aligned to its N. caninum ortholog NCLIV_001740 using ClustalO 1.2.4. Predicted features are shown above their corresponding sequences. Regions chosen for the deletion series are highlighted. (PDF) [file ppat.1012411.s002.pdf]

```
TGGT1_294860     MGRSDLIETCCRLQRSCKDLQCSLDELRDEGARLGAEKADLEREVAQTELLLERLHRDDGVVGNFFKDIGIHQISDFIQHVRTQTPEAAAAAAAAAAAAAAAAFGSSASALMKETQKHQFG    120
NCLIV_001740     MGRHDLIETCCRLQRSCKDLQCSLDELHDEDARLRAERTDLERQVAETQVLLERLHRDDGVVGNFFKDIGIHQVSDFIQQVRTQTPEAAAAAAAAAAAAAAVAFGSSASALMKETQKHQFG    120
                 *** **********************:**.*** **::****:**:*::**********************:*****:********************.******************

TGGT1_294860     IRREPVLDGLSRRQAAGLREQGQ-PVFQATQRKGEALLSSAERAASD-SIFTTASSSSLAGKTPTEAAKEIARAMTEAATEAWHRTSSQRLGLAGAGEKKAHKEEAEKASPLLESEEKQE    238
NCLIV_001740     VRRDALPDRHGRLEAGGFPCGKDQRDNRPVNTTGDAFLNSRERVADVPENAQRTGSSSLSGKTPAEAAKEIARAMTEAATEAWHRTSSQRLGLEGAGEKTEEVEGKQTH-QEGEEKKEKE    239
                 :**: : *  .* :*.*:    :    : .: .*:*:*.* **.*.  .    :.****:****:*************************** *****. . *  :.    *.::::*

TGGT1_294860     ERSRQDPFSDELVMRAVGQQGGSSVRALLQRKQTEERLQRQIAQWLDDASSLSIEEVPRPSQAFPVPSLASGLTPALSTVTSYSSSGASLAVTPGVSLPVDPPRESEAVRGEPGCEAGAN    358
NCLIV_001740     GRNWQDPFSDELVMKAVGQQGGSSVRALLQRKQTEERLQRQIAQWLDDASSLSIEEVPRPSGAFPLPGLASGLTAAPPSVPFSSSSLAASGVSPGVCMDGAQPTDSQAGSGESVCAEGDG    359
                  *. **********:********************************************* ***:*.****** *  :*   *** *: .*:***.:    * :*:*  **  *  * .

TGGT1_294860     SEKATQEGPAEVFEVEPAGADVPLSTHEERQRARQLCDARLLAMQVEKALGMLDIGRFAPQYLPSAACSAPADAYSTQKKDGSAPGEGDS-RAEGKRDGDACSGFIDGSSLDDLCARVFG    477
NCLIV_001740     GKAAQDTASNLAQAGGQGDGPSTLSAEEERQRARQLCDAKLLAMQVEKALGMFDIGRFAPQYAPVAA---PAASYSLLKGDASARPHGERAPDRTNRDRDAWSGFADG-SLDDLCARVLG    475
                 .: * : .   .    ...   **:.************:************:********* * **   ** :**  * *.**  .*:    . :** ** *** ** *********:*

TGGT1_294860     EQRNDTVPQIEAPQREKLRPQKERQDSKADSDKDAVEMKKELHKRGTASCVDAGSRGG-GGVLETKSSKENDEGQESERSPLCEGAASPAESQAEEQ--PSDANGLSLQPSSQEDGGFYA    594
NCLIV_001740     GNAQTKKAPGS-QQKKQRSPETGGLRSKA--DAETVEE---AQETGGAN-SDAMSRGDAPSLLEAKSNRAGVESDSSLPSRLCQAGLTSAQSPAPSQKLPPREDFPPSQPPREEEARASP    588
                  : : .    .  *::: *:.    *** * ::**     :: * *.  ** ***.  .:**:**.: . *.:.*  * **:.. : *:* * .*  *   :    **  :*:.

TGGT1_294860     ASSELREDGDGLETSSP--E---KTKIVFEREKPREEGREPASVPPAEQAETPSAVHALAPASVTWEGPPALGARGKKEEAEIGRLFLEEAHGDTTTGGAPGASKSRNETASLPASSSPF    709
NCLIV_001740     --APIIRSREGEEACVGQATRSSCCMQLSECESSQEEAAETASVPHTGQAAGPTKALDALAQTHSPHQDPGLGVKGEGEVAKEESSLK-ETLADTAPQVGCVDSPKSNATSAQSASKSCF    705
                   : : .. :* *:.            : * *. :**. * **** : **  *: .      : : .  *.**.:*: * *:    :  *: .**:    .   * . * *::  **.* *

TGGT1_294860     SPASAMTVECQLLPDGAVFLSWKFGPEPRALVAAAPAGTCRQLEVQVSVLDLTAQFEAASLKVSSSKTASPSGLLAPMQKTRAATLLLDSLQLEREYHLTLIARCMQLTFSTGVEEDCGE    829
NCLIV_001740     SPSSPMTVTCKLLPDGAVFLSWQFGPEPRTLLASAPPGACRQLEVQMSILDLTAQFEAATMRS----AAAPSGIQPPMQKTRASTLLLESLQLGREYHLTLIVRCLEFTFSTGVEEDCGA    821
                 **:* *** *:***********:******:*:*:** *:*******:*:**********:::     :*:***:  *******:****:**** ********.**:::***********

TGGT1_294860     PVCGVTVHFRLHADGLRSLRMRPVSLSTLAF-FPT--RRHAAPELPASTPHNAPAPTSYHSPSLSNGGNGSSAVTDQEYCSASSSPPASSSLASSSSSFVASSFARASAVRQKGAGQPVQ    946
NCLIV_001740     PVCGVTVHFRLHADGLKSLRMRPVSLSTLAFPFPATRRQVAAPELPANPTTPATLAPPAH-PSVPCKEENATFLAGQLD---PSSPAAPDRVS----------AASASPSGRDASAPPAL    927
                 ****************:************** **:  *: *******.    *      * **:    :.:: ::.*      *** * . ::           * **   :..:. *.

TGGT1_294860     PPCAHVDGEIVRADELRGVACLPEMKTPFSQQAVFSPSSARAESDVPRS-AEERVQSEAPGKAPVGCIETRPT--NCVPAAETSSLSQWTSTDESPCWRASSPNEFFFSPIPTDAGCTNA    1063
NCLIV_001740     TPCAALGGAGRARESESCGDLSRGLRAPTIQNVVCSLSSSRADREVDQKDAQERDGGSGLGRSTCSCTDVEAGQGRSVP-NGGSSLSLAVSTPAGTPWRACPGDDFLVRPVPGRAGHGPE    1046
                  *** :.*     :.          :::*  *:.* * **:**: :* :. *:**  ... *::  .* :..    ..**    ****  .**  .  ***.  ::*:. *:*  **

TGGT1_294860     RTQ---VAGFASHPTPPETTAFHSVAARPLSAASDTRGLLT-------------VAFQSPEGERVGPVGGEKGGTRSFSPSGSEEV----TLSTGRSEVGVEARPRTLGVSPLGRENISR    1163
NCLIV_001740     ERSRRAVEAALTTKLQAESAPFLSVAARPLCGPTGGLGVPAPPAADSPLTVAASVAPQEPEAEIRG------GGPAESVPPGSAALGSVELLSSSGSKAGAVRCRRVLGVAGKGERPVCS    1160
                 . .   * .  :    *:: * *******.. :.  *: :              ** *.**.*  *      **  .  * **  :     **:. *:.*.    *.***:  *.. :.

TGGT1_294860     SAFPHSSFGVSRPSPQLEPTSH-APSVGLPVAGSAGGTLGSVLPVFQAPGYPSGVRDNLPSPLHPSSVSAVSSSVLFFPQPAQGNLVPHATRLEARASPQSPSAP----TVCLDSAPESL    1278
NCLIV_001740     VSRHAADERFTR-VSGMEPSSLSSRLLGQPAVASA--ACPGSRPLSQASPAYAGVGDRLPSSFRASPLSAVSSSALF-PHTWQSSLSQSASP-ESSPGPQSQGSATHEGRGLDPAAPETR    1275
                  :   :.  .:*    :**:*  :  :* *...**  :  .  *: **    :** *.*** :: * :******.** *:  *..*   *:  *:  .*** .:          :***:

TGGT1_294860     PLARSTAALSDVVESLKSFE--ASAWQLARGGARAHQATLDGGEAS-------------SPSRLRGRGL-TSLGCLYTRT-DTEQGEEAERSDASAFRTSQEPGDACAAPGVSGCRLGGS    1381
NCLIV_001740     PVAGASVSLSEAVVSLKNLDVSDAAWALVRGVAQASQAALESAEASGLAGGIGGCGRGHATQGVRGRGLHESLGCLHARRTDADEGPEAETDERSLLS------ARLPAPGESTSHLGAS    1389
                 *:* ::.:**:.* ***.::   :** *.** *:* **:*:..***             : . :*****  *****::*  *:::* *** .: * :           *** * .:**.*

TGGT1_294860     LGSAAKASS--LGAEGGGRV-RSSSFRREQKQQWVQCRKQLWAMPESVRVRALSSSTSKTLSAGEAHQPAATPALQPNADFAAQGPTVESRLPGSSPVAPQSGSSDADSLASGLPCGSPS    1498
NCLIV_001740     LGSAAPARGPCGGNAPDGRVRRSSLSRREQKKHWIQCRKQLWAMPDSIRVLALNPSAGT-VASGEATQPSVREQPV------GRRP-AVSAVPGSVSA--QSCLPN-IDASRSSSFGSPS    1498
                 ***** * .   *   .*** ***  *****::*:**********:*:** **. *:.. :::*** **:.            .: * . * :***  .  **   :  . : .   ****
```

```
TGGT1_294860      LHVPSLGQGSDFSQGNAPLSHAVRSWPPTEETNAHPGKKNLFPETATDAASPPVAEV------------LHRGGTYAAEAFAAAQSPATVSSTAPVSEGSGAKRLNPSRGATRDLEASVP      1606
NCLIV_001740      LHVPSLGQGSTSAQSGDPAQAESSLDL---------------SSAVSDVREPPRGVTASNLPSCSSERAERRKASFRVA-------PCA------LSEG--PPHLS--HGE---L-----      1578
                  **********  :*.. * .                      .:.:*. .** . .              :* .:: .        *.:      :***    :*.  :*    *

TGGT1_294860      GAQDTCRHPEARLTTGGVAPAATSASARDVFGLEAEKSRVRRENETDGPNPTVYEVGALASLMAVRRQEEELKKVLQEQRVKQQQLQRRQQDLQRHLEAAGIREQRLQASHSSLSGAATA      1726
NCLIV_001740      --RAETAHGRAQVTAGGSESQ--------AVGPAGEN--PVNRGSDGRPAPGASEVDALASLRALRQQEEEITKLLRAHRDEQLQLQRRRMDLQGHLAGGAAPDTRVRSSRSCPFGEGAS      1686
                    :    * .*::*:**            ..*  .*:    .... . * * . **.***** *:*:****:.*:*: :* :* *****: *** **  ...  : *:::*:*.  * .::

TGGT1_294860      PESDVVSVKSGVSRDASDRRPTQAAGGECSGMSACGTSLETRVGAGEPGEGRDQDSREGEKSLRGDPPQMPKVVGSLAVKAAP--PANLSVIAPKTQTHREAAASLSSSPSRAAQSCLLS      1844
NCLIV_001740      ----------------ERGPVLGTAG-------------------AASESEQSGREGQMSST-GRPQTPKVVGSLTVMKAASPPGNPG-GDPAAL----TFLPLAASPPLGTNRLSLS      1763
                                  :* *. .:.*                   ..  :*..***: *   . ** *******:*  *   *.* .   * :      :   *::**  .::   **

TGGT1_294860      GSVASGVFPNHRSCSVAPGVSL--HASEPRLYYANSLVSSREDLLTEATKHSGQVTNMVSSSQEILRAGESGVFAVATDLEKRQEAHQGAHPRVQGLSLQSFPPCGFQDIPAGGR--ERL      1960
NCLIV_001740      GPVSSAASSAPNPCVAAPAATAAPHYPQPAHYAG-NLGATR-SLARVGTEASRALVTSSVSSQGLPRAGDCGASAAGREPEKRAGGNGEERPS-GGE--------GLHGGLAGERSTGAG      1872
                  * *:*..     . * .**..:   *  :*  * . .* ::* .*   .*: *  :..   *** : ***:.*. *.. : ***  .:   :*   *         *::.  ** *

TGGT1_294860      GGAVGVPTALPVVGLGKNVSLLDGKRMRYAGATQSSPQQLWAPELGAPLPRVPPLDPQMAQAAPRRLSPNRRPTAGDTSRTLPSPWSTPQRMHRAGFPNAPAAPAAASPLANRDRAARTP      2080
NCLIV_001740      GGSLGPTTRPHSVRVQLHADSQAGRRVRYAGAPQCFPQQLWAPELGVPVPQIPPLDSTAC-AAPYRLPS-RQPTSGDASRALPPGSS--QR-HGVGFPGAAS-VSVDSPLASSDRSARTP      1986
                  **::*  *    * :  :..    *:*:***** *. **********.*:*::****    . *** **   *:**:**:**:**   *  ** * .***.* :  :. ****. **:****

TGGT1_294860      STETGLRRVPLGDGG----DGATGQSSGEKQVKETQQTDTEASWGSRVLTSLGSAVLALSPSRSSALSQASVSLQASTSLQTRRRSLEPVGRVDGRPSAALLPPELNASASPSLF-----      2191
NCLIV_001740      SAAAVH-RFAGGSGGGTAGNGAAGKAPVEDH-VDRKRRESEASWGSRVLTSLGSAVSAFSPSRASASS------------QTRHRSLEPPSAFEGEQKSARVPPGADASVSRRLSTATPA      2092
                  *: :   *.  *.**    :**:*::  *.:  : :: ::**************** *:****:** *            ***:***** . .:*. .:* :**  :**.*  *

TGGT1_294860      -------AAASPPRSVAADPRLMHTMSLSIQPMPR-GIL-APQGNQAFP-GLPLVPANAPAPRP-VAAASSVSGVSVFPSPGVVSPSV-TASFSSAAQVLPGNALGGAVHPQCSGFGLLR      2299
NCLIV_001740      PRGPQAPAASPPLFSAAADPRLLHTLSLSRQQIPPRGLFVMPHGNQVVQCPLPSATQSVPASRPPGAAAPSFSGVS-LPSPGVSPPPAGPSSLSGAPQMYTRRTLGGPVYPQHPAFTGIH      2211
                         **: *  *.******:**:*** * :*  *::  *:***..   **  .  ..** **  *** *.**** :*****  *  .  :*:*.* *:   .:*** *:**  .*  ::

TGGT1_294860      PAS-GA---PSSPVVSAARLPPGASAVHTPKALT--SGVARPAPLSYAHHLAVTSQNSGVAAAASLAVPGDGKENRGTHEAAEVTRLQETAP---------GAQAVQAPVYKPASFVSLS      2404
NCLIV_001740      RQAPALPEVSSPQALSADGLPPGAFAVTTLLQNGGASVSSRSPFPPYAHRLTLTPQRN-EQRDASLSVRGDGGKNHAKREGGEVTRLTLPSTCVGGYQSAVVAQGAKTPFFKPASCESRA      2330
                   : .     *  .:**  ***** ** *        *  :*     ***:*::* *..     ***:* *** :*:..:*..*****   :             **..::*.:****  * :

TGGT1_294860      QGEA-REGPGSQSANAVSAKGGDTAGHSGTAQLLRPSSGPPGGAGKLGRQVTFY      2457
NCLIV_001740      PREEAIVSTDSKRINPVSAKAAG--RPGGAVQLPARDSSPVVAAGGLGRQVTFY      2382
                   *    . .*:  * ****...    .*:.**   .*.*  .** ********
```

Predicted Features:

- Coiled-coil
- Helix
- Strand
- Myristoylation
- Palmitoylation

Residues selected for deletions:

- 2-169
- 170-375
- 376-569
- 570-700
- 701-877
- 900-1200
- 1201-1519
- 1520-1817
- 1818-2027
- 2028-2237
- 2238-2457
