# Supplementary material for: BCC0 collaborates with IMC32 and IMC43 to form the Toxoplasma gondii essential daughter bud assembly complex
Source: PLoS Pathog. 2024 Jul 18;20(7):e1012411. doi: 10.1371/journal.ppat.1012411 (PMC11288415; doi:10.1371/journal.ppat.1012411)
Supplement: Attachment — Submitted filename: BCC0-IMC32-IMC32 Response Letter.pdf [file ppat.1012411.s011.pdf]

Dear Peter,

Thank you very much for submitting your manuscript "BCC0 collaborates with IMC32 and IMC43 to form the Toxoplasma gondii essential daughter bud assembly complex" for consideration at PLOS Pathogens. As with all papers reviewed by the journal, your manuscript was reviewed by members of the editorial board and by several independent reviewers. The reviewers appreciated the attention to an important topic. Based on the reviews, we are likely to accept this manuscript for publication, providing that you modify the manuscript according to the review recommendations.

You will see that one of the experts who reviewed your manuscript mentions the somewhat incremental nature of the work, which is something I initially quite agreed with. On the other hand, all three reviewers found that it is a thorough and very complete study, and thus in the end I feel that, overall, the insightful information it brings about the assembly of the inner membrane complex would justify publication in PLOS Pathogens. However, before the manuscript can be formally accepted you should address carefully all the points raised by the reviewers. I also ask you to please consider the following additional points:

- at the end of the Summary (l. 54-55), as the present work does not provide direct insights into the development of new anti-toxoplasma drugs, I think that the "which could lead to the identification of drug targets" part should be removed.

We removed this phrase as requested.

- on l. 193 you mention a "93% reduction in the level of BCC0 expression", which implies that some quantifications have been performed, yet they are not shown in the manuscript. Either show them or remove this particular number.

We added a graph showing the western blot quantification to Figure 3C. The raw data for the quantification can also be found in new Table S4.

Please prepare and submit your revised manuscript within 30 days. If you anticipate any delay, please let us know the expected resubmission date by replying to this email.

When you are ready to resubmit, please upload the following:

[1] A letter containing a detailed list of your responses to all review comments, and a description of the changes you have made in the manuscript.

Please note while forming your response, if your article is accepted, you may have the opportunity to make the peer review history publicly available. The record will include editor decision letters (with reviews) and your responses to reviewer comments. If eligible, we will contact you to opt in or out

[2] Two versions of the revised manuscript: one with either highlights or tracked changes denoting where the text has been changed; the other a clean version (uploaded as the manuscript file).

Important additional instructions are given below your reviewer comments.

Thank you again for your submission to our journal. We hope that our editorial process has been constructive so far, and we welcome your feedback at any time. Please don't hesitate to contact us if you have any questions or comments.

Sincerely,

Sébastien Besteiro, PhD

Academic Editor

PLOS Pathogens

Meera Nair

Section Editor

PLOS Pathogens

Michael Malim

Editor-in-Chief

PLOS Pathogens

orcid.org/0000-0002-7699-2064

****

Reviewer Comments (if any, and for reference):

Reviewer's Responses to Questions

Part I - Summary
Please use this section to discuss strengths/weaknesses of study, novelty/significance, general execution and scholarship.

Reviewer #1: This study reports on dissecting the organization and function of a complex of three early recruited daughter proteins which are essential for the proper assembly of the IMC during T. gondii tachyzoite endodyogeny. The work is thorough and complete in all aspects, making this a very robust report on how this complex interacts with each other, and which sections of BCC0 are essential to its function. The elegant domain dissection using a series of deletion constructs in different background strains highlights only the domain identified by Y2H for interaction with IMC32 is essential for localization and completion of the lytic cycle. As discussed by the authors, it is somewhat surprising none of the acylation sites exerted a critical function, but the data is clear and convincing and indeed as remarked, these are only predicted acylation sites. Lastly, the domains on both IMC32 and BCC0 that are interacting with each other were exhaustively mapped using pairwise Y2H with deletion mutants. As the authors discuss, many questions remain as to the regulation of this complex in space and time, but this study lines up the tools to tackle these questions next. In all, the study is a detailed report on a critical complex key to the initiation of daughter budding and therefore entails a major step forward in our understanding of this process unique to apicomplexan parasites

Reviewer #2: In this study, Pasquarelli et al. investigate the organization of the inner membrane complex (IMC) during the internal development of the daughter cells in Toxoplasma gondii. Capitalizing their previous work on IMC32 (PMID: 33593973) and IMC43 (PMID: 37782662), they dig further into the composition and function of the complex in which these two proteins are involved. Crossing data from a yeast two-hybrid screen and proximity labeling, they identified BCC0 as a third component of this “essential daughter bud assembly complex”. They convincingly show that BCC0 recruitment is dependent of IMC32 and IMC43. Using a complementation strategy with a large collection of BCC0 mutants, they pinpoint the region of the protein essential for both its localization and function. Surprisingly, only the construct lacking the region 701-877 has the same impact as the depletion of the full-length protein. A contribution of residues 170-375 was also observed and the authors then assessed how much of this fairly large protein is sufficient for its function. They show that the C-terminal part of the protein, downstream of residue 899, is dispensable.
Finally, using previous Y2H constructs of IMC32 and IMC43, the authors identified the regions involved in the direct interactions of the proteins and showed that BCC0 and IMC43 bind to IMC32 leading to the proposed model of IMC assembly with the hierarchical recruitment of IMC32, IMC43 and BCC0.
The manuscript is clear and well-written. The experiments are well-conducted and controlled. The IMC is a crucial component of the daughter cell assembly in Apicomplexa and its components not present in the host could constitute an attractive

drug target. While these proteins are not conserved across the phylum but found in Coccidians, the manuscript is, to my view, of interest for the audience of PLOS pathogens. It contributes to the elucidation of the molecular assembly of daughter cell IMC. I just have a few minor concerns/corrections about the paper.

Reviewer #3: The manuscript by Pasquarelli and colleagues investigates the role of IMC32, IMC43, and BCC0 for Toxoplasma gondii inner membrane formation. Using proximity labeling and Y2H, the authors demonstrate a likely complex of IMC32, IMC43, and BCC0. Straightforward reverse genetic experiments demonstrate that BCC0 is essential and that 701-877 are critical for localization and function of the protein. The studies are well done, and the data are convincing. The manuscript provides a new level of knowledge about IMC biogenesis, an area of apicomplexan biology that is really lacking many details. Thus, the study provides an important, albeit somewhat incremental, step forward in our understanding of endodyogeny.

We thank the reviewers for their positive comments.

Part II – Major Issues: Key Experiments Required for Acceptance
Please use this section to detail the key new experiments or modifications of existing experiments that should be absolutely required to validate study conclusions.

Generally, there should be no more than 3 such required experiments or major modifications for a "Major Revision" recommendation. If more than 3 experiments are necessary to validate the study conclusions, then you are encouraged to recommend "Reject".

Reviewer #1:

1. Fig 5E reports on plaque size, but another aspect was the sharp reduction in plaque number reported in Fig 3D. How did the complementation with delta701-877 affect the number of plaques? It would be great if this could be included.

As requested, we have added a graph showing the plaque efficiency (# of plaques formed divided by the # of parasites infected) in new Figure 5F. Just like with plaque size, the BCC0^{cKD} + BCC0$^{\Delta 701\text{-}877}$ strain completely failed to rescue the defect in plaque number/efficiency.

Reviewer #2:

(No Response)

Reviewer #3:

Major points (although none are really major):

1. Fig 3D vs 5E plaque size for BCC0 cKD are somewhat different. Is this difference meaningful or merely showing the variability of biological assays? This should be stated either way.

In Figure 3D, treating $BCC0^{cKD}$ with ATc caused a 92% reduction in plaque number/efficiency and an 80% reduction in plaque size. In Figure 5E-F, we showed an 83% reduction in plaque efficiency and a 62% reduction in plaque size. These plaque assays were conducted months apart using different batches of host cells, so it is possible this is due to the normal variability in biological assays, as suggested by the reviewer. Alternatively, it is possible that the leaky expression of $BCC0^{cKD}$ parasites, which we documented in Figure 3C, became more severe over time. We added a few sentences commenting on this in lines 241-245 of the marked-up manuscript.

Regardless, the plaque assays in Figure 5E were meant to compare the phenotype of $BCC0^{cKD}$ parasites with $BCC0^{cKD}$ + $BCC0^{\Delta 701\text{-}877}$. As these two strains phenocopy each other, we believe the data presented is still sufficient to draw the conclusion that residues 701-877 are essential for function of BCC0 despite the slightly milder growth defect compared to Figure 3D.

2. It would be helpful to have a the cKD control included in figure 6J. This would be helpful so that a comparison can be made between the "partial" complementation and no complementation. Alternatively, it would be ideal to have some sort of inducible knockout for endogenous BCC0 with each of the complementation strains. That would allow for immediate removal of wt BCC0 without the ability for compensatory mutations to occur. This is mostly technical because the results are quite robust, as is.

Figure 6J shows the phenotype of the wild-type $BCC0^{smOLLAS}$ strain compared to Δ*bcc0* parasites complemented with $BCC0^{WTc}$, $BCC0^{170\text{-}899}$, $BCC0^{376\text{-}899}$, $BCC0^{570\text{-}899}$, and $BCC0^{701\text{-}899}$. None of these mutants were assessed in the context of the $BCC0^{cKD}$ strain, thus the $BCC0^{cKD}$ strain would not be an appropriate control in this experiment. Furthermore, the incomplete knockdown of the $BCC0^{cKD}$ strain would make assessing the partial complementation of these mutants much more difficult.

Redoing the deletion series in a BCC0 inducible knockout strain would require substantial work (generating and validating the inducible KO strain, complementing the inducible KO strain with all 16 mutant constructs, and performing IFAs and plaques assays, plus quantifications, for each of these new strains). As the reviewer stated, the results are quite robust already and these new experiments would not change the conclusions of the manuscript.

3. It would be interesting for the authors to hypothesize or at least discuss how the BCC0 truncations, especially the one that is just 701-899, partially complements despite mislocalization. The discussion mentions the interaction with IMC32 as the driver. However, this seems like it is likely to allow the truncated protein to interact but not how it functions without 80+% of the protein. This is sort of present already in lines 300-318. However, the results still seem surprising.

We agree that this particular result is surprising. We added the following statement to the discussion (lines 317-320 in the marked-up manuscript) to address this more fully:

“Even more surprisingly, expression of just the essential domain of BCC0, residues 701-877, was sufficient for parasite viability, although the protein was severely mislocalized and these parasites exhibited defects in growth and morphology. This suggests that a small percentage of $BCC0^{701-899}$ is able to localize to the daughter cell scaffold and provide sufficient function to support viability. “

Part III – Minor Issues: Editorial and Data Presentation Modifications
Please use this section for editorial suggestions as well as relatively minor modifications of existing data that would enhance clarity.

Reviewer #1:

1. Plaque assay quantifications. Although the M&M states 30-50 plaques were measured for size, it does not state whether biological reps were performed. In the Fig legends it does not provide details either. Please also add whether the mean/average/median is plotted and what metric the error bars represent. To be complete, inclusion of all quantitation and analysis data as a supplement is recommended.

A statement about biological replicates can be found in the materials & methods section (line 505 in the mark-up file, “All plaque assays were performed in triplicate.”) We added lines to this section about what values were plotted and noted that the error bars represent standard deviation. We also reported all of the raw data for plaque assays in new Table S4.

2. Fig 1B. The apicoplast is known to harbor biotin, yet in the -biotin control panel no streptavidin signal is visible at all. Please comment.

The streptavidin AlexaFluor 594 we were using was an old tube that was highly sensitive to photobleaching. We went back to our original IMC32 TurboID IFA and captured a new -biotin control image where the apicoplast is visible (see replaced image in Figure 1B).

Reviewer #2:

I have only a few minor concerns about the paper:

1. As mentioned in the text (line 209), the authors used an alignment of the apicomplexan BCC0 and secondary structure predictions with PsiPred to establish all the constructs described in the study. It would be useful to see this sequence homology as a supplementary figure to better understand how the constructs have been chosen and how much the region between residues 1-899 is conserved.

As requested, we added a new supplemental figure (new Figure S2 – note that this changed the numbering for the other supplemental figures) which shows an alignment between the amino acid sequences of BCC0 (TGGT1_294860) and its *N. caninum* homolog (NCLIV_001740). Predicted features from PSIPRED, DeepCoil2, CSS-Palm, and GPS-Lipid are shown above their corresponding sequences. Regions chosen for the deletion series are highlighted.

2. The protein TGGT1_280370 is ranked A from the Y2H screen and is the 2nd protein enriched of the cross between the Y2H and the proximity labeling. Did the author assess its localization? It might be out of the scope of this suty.

We did not explore TGGT1_280370 due to its predicted nuclear localization by hyperLOPIT (Barylyuk et al. 2020), relatively modest phenotype score (Sidik et al. 2016), and significantly different transcriptional profile throughout the cell cycle compared to IMC32 (Behnke et al. 2010). Since we were searching for essential components of the daughter IMC, we chose not to pursue this candidate binding partner and to focus instead on BCC0.

3. Figure 4. The panel with the plaque area graph should be panel P and accordingly, line 227 “Figure 4M” should be “Figure 4P”.

Thank you for pointing out this mistake. We corrected this.

4. In the proposed model, how to place IMC44 that interacts with IMC43?

In our previous work, we demonstrated that IMC44 binds to IMC43. However, since IMC44 is dispensable and we are focused on the essential components of the daughter IMC, we did not study it any further. We have not assessed whether IMC44 interacts with either IMC32 or BCC0, which is why we did not place it in the model at the end of this paper.

Reviewer #3:

Minor points:

1. Line 67 – this notes that current therapies for Toxoplasmosis are not well tolerated in humans. This is not really true because the first line medications, pyrimethamine with sulfadiazine, is actually very well tolerated when dosed properly. The statement does not add to the manuscript and is frankly incorrect.

We removed this statement as requested.

2. Line 198-200. It would be helpful for the reader to know what the tags were for IMC32 and IMC43. This should be added to the text here.

We used a 3xMyc tag for IMC32 and an smHA tag for IMC43. We added this information to the text as requested.
